# Automated computer quantification of breast cancer in small-animal models using PET-guided MR image co-segmentation

**DOI:** 10.1186/2191-219X-3-49

**Published:** 2013-07-05

**Authors:** Ulas Bagci, Gabriela Kramer-Marek, Daniel J Mollura

**Affiliations:** 1 Center for Infectious Disease Imaging, , Bethesda, MD 20892, USA; 2 Radiology and Imaging Sciences, National Institutes of Health, Bethesda, MD 20892, USA; 3, The Institute of Cancer Research, London, UK

**Keywords:** Image segmentation; Computer quantification; FDG-PET; MRI/PET; Breast cancer; Small-animal models; Co-segmentation; Volume quantification; Random walk

## Abstract

**Background:**

Care providers use complementary information from multiple imaging modalities to identify and characterize metastatic tumors in early stages and perform surveillance for cancer recurrence. These tasks require volume quantification of tumor measurements using computed tomography (CT) or magnetic resonance imaging (MRI) and functional characterization through positron emission tomography (PET) imaging. *In vivo* volume quantification is conducted through image segmentation, which may require both anatomical and functional images available for precise tumor boundary delineation. Although integrating multiple image modalities into the segmentation process may improve the delineation accuracy and efficiency, due to variable visibility on image modalities, complex shape of metastatic lesions, and diverse visual features in functional and anatomical images, a precise and efficient segmentation of metastatic breast cancer remains a challenging goal even for advanced image segmentation methods. In response to these challenges, we present here a computer-assisted volume quantification method for PET/MRI dual modality images using PET-guided MRI co-segmentation. Our aims in this study were (1) to determine anatomical tumor volumes automatically from MRI accurately and efficiently, (2) to evaluate and compare the accuracy of the proposed method with different radiotracers (^18^F-Z _HER2_-Affibody and ^18^F-flourodeoxyglucose (^18^F-FDG)), and (3) to confirm the proposed method’s determinations from PET/MRI scans in comparison with PET/CT scans.

**Methods:**

After the Institutional Administrative Panel on Laboratory Animal Care approval was obtained, 30 female nude mice were used to construct a small-animal breast cancer model. All mice were injected with human breast cancer cells and HER2-overexpressing MDA-MB-231HER2-Luc cells intravenously. Eight of them were selected for imaging studies, and selected mice were imaged with MRI, CT, and ^18^F-FDG-PET at weeks 9 and 10 and then imaged with ^18^F-Z _HER2_-Affibody-PET 2 days after the scheduled structural imaging (MRI and CT). After CT and MR images were co-registered with corresponding PET images, all images were quantitatively analyzed by the proposed segmentation technique.

Automatically determined anatomical tumor volumes were compared to radiologist-derived reference truths. Observer agreements were presented through Bland-Altman and linear regression analyses. Segmentation evaluations were conducted using true-positive (TP) and false-positive (FP) volume fractions of delineated tissue samples, as complied with the state-of-the-art evaluation techniques for image segmentation. Moreover, the PET images, obtained using different radiotracers, were examined and compared using the complex wavelet-based structural similarity index (CWSSI). (continued on the next page) (continued from the previous page)

**Results:**

PET/MR dual modality imaging using the ^18^F-Z _HER2_-Affibody imaging agent provided diagnostic image quality in all mice with excellent tumor delineations by the proposed method. The ^18^F-FDG radiotracer did not show accurate identification of the tumor regions. Structural similarity index (CWSSI) between PET images using ^18^F-FDG and ^18^F-Z _HER2_-Affibody agents was found to be 0.7838. MR showed higher diagnostic image quality when compared to CT because of its better soft tissue contrast. Significant correlations regarding the anatomical tumor volumes were obtained between both PET-guided MRI co-segmentation and reference truth (*R*^2^=0.92, *p*<0.001 for PET/MR, and *R*^2^=0.84, *p*<0.001, for PET/CT). TP and FP volume fractions using the automated co-segmentation method in PET/MR and PET/CT were found to be (TP 97.3*%*, FP 9.8*%*) and (TP 92.3*%*, FP 17.2*%*), respectively.

**Conclusions:**

The proposed PET-guided MR image co-segmentation algorithm provided an automated and efficient way of assessing anatomical tumor volumes and their spatial extent. We showed that although the ^18^F-Z _HER2_-Affibody radiotracer in PET imaging is often used for characterization of tumors rather than detection, sensitivity and specificity of the localized radiotracer in the tumor region were informative enough; therefore, roughly determined tumor regions from PET images guided the delineation process well in the anatomical image domain for extracting accurate tumor volume information. Furthermore, the use of ^18^F-FDG radiotracer was not as successful as the ^18^F-Z _HER2_-Affibody in guiding the delineation process due to false-positive uptake regions in the neighborhood of tumor regions; hence, the accuracy of the fully automated segmentation method changed dramatically. Last, we qualitatively showed that MRI yields superior identification of tumor boundaries when compared to conventional CT imaging.

## Background

Early detection and characterization of breast cancer, combined with an accurate estimation of tumor volume and shape, and metabolic information can help predict the burden and the severity of the disease. For this purpose, non-invasive anatomical imaging methods such as magnetic resonance imaging (MRI) and computed tomography (CT) are widely used in the clinics to obtain high-resolution anatomical information about a patient’s breast cancer status. Positron emission tomography (PET), on the other hand, is often used in conjunction with CT and more recently with MRI [1] to provide the molecular process of cell/tissue activity information from cancer sites via specific radiotracers [2]. Among all of them, the most frequently used is the radio-labeled glucose analog, ^18^F-fluorodeoxyglucose (^18^F-FDG). However, ^18^F-FDG lacks the specificity to identify the receptor status over-expressed in breast cancer because it reflects the metabolic activity of cells. Since glucose metabolism is not specific for malignant processes, physiologic ^18^F-FDG uptake occurs in normal tissues (brain, muscles, salivary gland, myocardium, and urinary tract) and is also taken up by various inflammatory and benign lesions, which could potentially lead to false-positive or negative findings [3,4]. ^18^F-Z _HER2_-Affibody, in contrast to FDG, is shown as a promising radiotracer for the characterization of HER2-positive breast cancer metastases because it characterizes HER2-positive lesions with higher precision than ^18^F-FDG [3]. However, even when an appropriate radiotracer is chosen to monitor functional changes in cancerous tissues, morphological measurement of tumor volume with CT or MRI still remains a challenging task. Similarly, quantitative measurements of radiotracer activity for a region of interest (ROI) are prone to errors. For example, the severity of disease can easily be underestimated because of the errors due to inappropriate ROI definition or inaccurate delineations. Also, the overlap or close juxtaposition of the abnormal signal with the surrounding normal structures and the background radiotracer activity are other source of errors affecting the quantification process significantly [5].

Much of the relevant literature regarding the quantitative analysis of metastatic breast cancer has relied on manual methods for image analysis with qualitative and/or semi-quantitative measurements due to the scarcity of automated computer-assisted tools for different imaging modalities [6-9]. However, manual approaches are highly time-consuming; thus, they consequently reduce the efficiency of research and have the lower reproducibility rates. Because of these reasons, developing an efficient computer-aided quantification tool that provides accurate and reliable anatomical tumor volumes, its extent, as well as metabolic activity estimations for HER2-positive tumors is highly desirable.

In this study, we proposed a fully automated PET-guided random walk image co-segmentation method for HER2-positive tumor volume quantification. Our aim was to design a reliable, reproducible, and efficient gross tumor volume estimation tool that could be used in clinical routine. Our proposed method first identified HER2positive tumors automatically from PET images. Second, it determined the extent of the detected tumor regions from corresponding anatomical images (either CT or MRI) using one-to-one voxel correspondence properties of the fusion process (MRI/PET or PET/CT) followed by segmentation of the tumor regions using both functional and anatomical images automatically. In our approach, after we estimated active gross tumor volumes accurately, we compared the results with radiologist-derived anatomical tumor volumes (i.e., reference standard). In summary, our primary endpoints in this study were to identify roughly the spatial position of HER2-positive tumors automatically from PET images and extract the boundary of the corresponding anatomical regions in high accuracy, to compare the computer-assisted volume quantification performances with PET/CT, with respect to the chosen radiotracers, and to test the performance of the proposed approach, with respect to the radiologist-derived volumes.

## Methods

All animal protocols were approved by the Institutional Administrative Panel on Laboratory Animal Care [10]. Thirty female nude mice were used in laboratory experiments. All mice were injected with human breast cancer cells (5.0 × 105, MDA-MB-231HER2-Luc) through the tail vein. Twenty-six mice developed lesions in the lungs, eight of them were selected to establish our imaging studies due to detectable metastasis progression, and four mice did not develop detectable tumors. Animals were imaged with MRI, CT, and ^18^F-FDG-PET at weeks 9 and 10 and then imaged with ^18^F-Z _HER2_-Affibody-PET 2 days after the scheduled structural imaging (MRI and CT). PET, CT, and MRI images were quantitatively analyzed by the proposed method. Tumor volumes from CT and MRI were obtained simultaneously and tracked longitudinally at the end of the 10 weeks. The results were compared to radiologist-derived reference truths.

### PET imaging technique

After the Affibody molecules were provided by a Cooperative R &D Agreement partner (Affibody AB, Stockholm, Sweden), they were labeled with ^18^F by the steps described in [11]. All mice were imaged on the Advanced Technology Animal Scanner (ATLAS, Bethesda, MD, USA) using both ^18^F-FDG and ^18^F-Z _HER2_-Affibody radiotracers. Animals were imaged 2 days apart at weeks 9 and 10, scanned in a prone position (5- to 10-min emission scans, two bed positions) with a 100- to 700-keV energy window. The transverse and axial field of views (FOVs) on the scanner were set at 6.8 and 2 cm, respectively. On scanning days, mice were fasted for approximately 4 h, and they were allowed to acclimate to the ^18^F-FDG-PET imaging facility environment for at least 1 h. After acclimation, mice were anesthetized using isoflurane/O_2_, followed by an injection of the radiotracer (7.4 MBq, 100 *μ*L for ^18^F-FDG) through the tail vein. All animals were imaged after a period of radiotracer uptake distribution (1 h) [12]. ^18^F-Z _HER2_-Affibody (6.6 to 7.4 MBq, 100 *μ*L) was also imaged 1 h post-infection but 2 days later. Note that the uptake was maximized 1 h after injection and preserved its maximized state for approximately 2 h when ^18^F-Z _HER2_-Affibody was used [13]. A calibration constant, obtained from scanning ^18^F molecules, was used to correct reconstructed images, which have no attenuation correction procedure.

### MR and CT imaging technique

MR images for mice were acquired on a Philips Achieva 3T clinical MR scanner (Cleveland, OH, USA), with a specifically designed receiver coil, typically used for small animals (44-mm diameter × 70-mm long). The fast field echo (FFE) parameters were as follows: repetition time (TR)=15.3 ms, echo time (TE)=2.4 ms, flip angle (FA)=20°, FOV=36 mm × 24 mm, resolution =0.19 mm × 0.25 mm, slice thickness =0.562 mm, number of slices =32, and scan time =7.65 min. In order to fully appreciate tissue boundaries, respiratory-triggered multi-slice T2-weighted MR images acquired from a turbo spin echo sequence was used; the parameters were as follows: TR=4 breathing cycles ≈4,400 ms, TE=65 ms, FA=90°, echo train length =11, FOV=36 mm × 24 mm, resolution =0.19 mm × 0.19 mm, slice thickness =0.562 mm, number of slices =32, and scan time ≈14 min. We also imaged the mice with the CT component of a NanoPET/CT scanner (Bioscan Inc., Washington, DC, USA) by setting the X-ray tube’s high voltage at 55 keV (sampling time =1,100 ms, in-plane resolution of pixels =78 *μ*m) to compare our proposed methodology with different anatomical modalities.

### Co-registration of multimodal images

PET, CT, and MRI images were acquired at different times, on the same day, and on different scanners. Locally affine globally smooth affine transformation with tri-linear interpolation [14-16] was used to spatially co-register PET images into anatomical correspondence (CT and MRI) in order to have one-to-one voxel correspondence. Images were also interpolated to provide the same number of slices between functional and anatomical images. Validation of the registrations was further assessed via visual inspection by three independent expert clinicians.

### Automated PET-guided random walk image co-segmentation

Our approach for delineating tumor boundaries in MRI is similar to the co-segmentation method used by Bagci et al. [17], except for one criterion: the PET image was ‘roughly’ segmented first in our approach. These rough segmentations were used as foreground seeds (clues) to identify the location of the tumors. This (identification) process is quite robust as we only selected the voxels with the highest intensity values as ‘foreground seeds’ within the roughly segmented regions. A simple 8-connected (in 2D) or 26-connected (in 3D) neighborhood search was conducted to identify background seeds by finding the closest voxels nearby the foreground seeds, pertaining to background regions. We next propagated all background and foreground seeds into the corresponding MR image to guide and finalize the delineation process in the structural image domain. This process can be named PET-guided random walk image co-segmentation, due to the guidance of the delineation by signatures from PET images. With this algorithm, voxels having no label were assigned probabilities based on the random walkers’ computed probability, measuring the strength of the path initiated from labeled signatures to reach the unlabeled voxels first. Note that high and low uptake regions in PET image were used as foreground and background signatures. The overall process was performed in three dimensions, and it took only a few seconds per lesion. The number of voxels enclosed by the boundary of the pathological site was used to estimate the volume of the cancerous tissues. We also conducted the same segmentation experiments on CT images in order to compare effectiveness of PET/MRI dual modality images to PET/CT.

It is also important to emphasize that in [17,18], we proposed a general co-segmentation framework based on simultaneous random walk on a space formed by fusing complementary information from PET and CT/MRI images. By introducing certain ‘visibility’ parameters (i.e., weights for PET and MRI or CT images), resultant delineations can be made much more precise. In this manuscript, we specifically set the visibility parameter of the PET images much lower than the visibility parameter of the corresponding anatomical images so that the resulting delineation was performed on the anatomical images. The reason behind this was because the tumor extent was observed better in MR images than PET images.

When MRI and PET images of the same subject are co-registered prior to the segmentation process, there is *one-to-one voxel correspondence* between them. Once this correspondence is established, the next step in our algorithm is to detect a ROI from the PET images. Defining a ROI can be fully automated or semi-automated. In this work, we used seed-based identification of the significant uptake regions. The significant uptake regions (i.e., foreground) were found roughly through finding voxels with the ‘maximum intensity’ value. Those voxels were considered as foreground cues (seeds). We provided identification of foreground seeds by defining an encoder function *c*(*λ*), equivalent to thresholding for PET images as:

(1) $$c(\lambda )=\left\{\begin{array}{cc} 1, & \lambda \in \left[{\text{SUV}}_{\text{max}}/N,{\text{SUV}}_{\text{max}}\right],\\ 0, & \text{otherwise,} \end{array}\right)$$

where (N≥1.5)∈R is a free parameter and SUV stands for standardized uptake value. This process was followed by background identification (i.e., background seeds).

**Figure 1:**
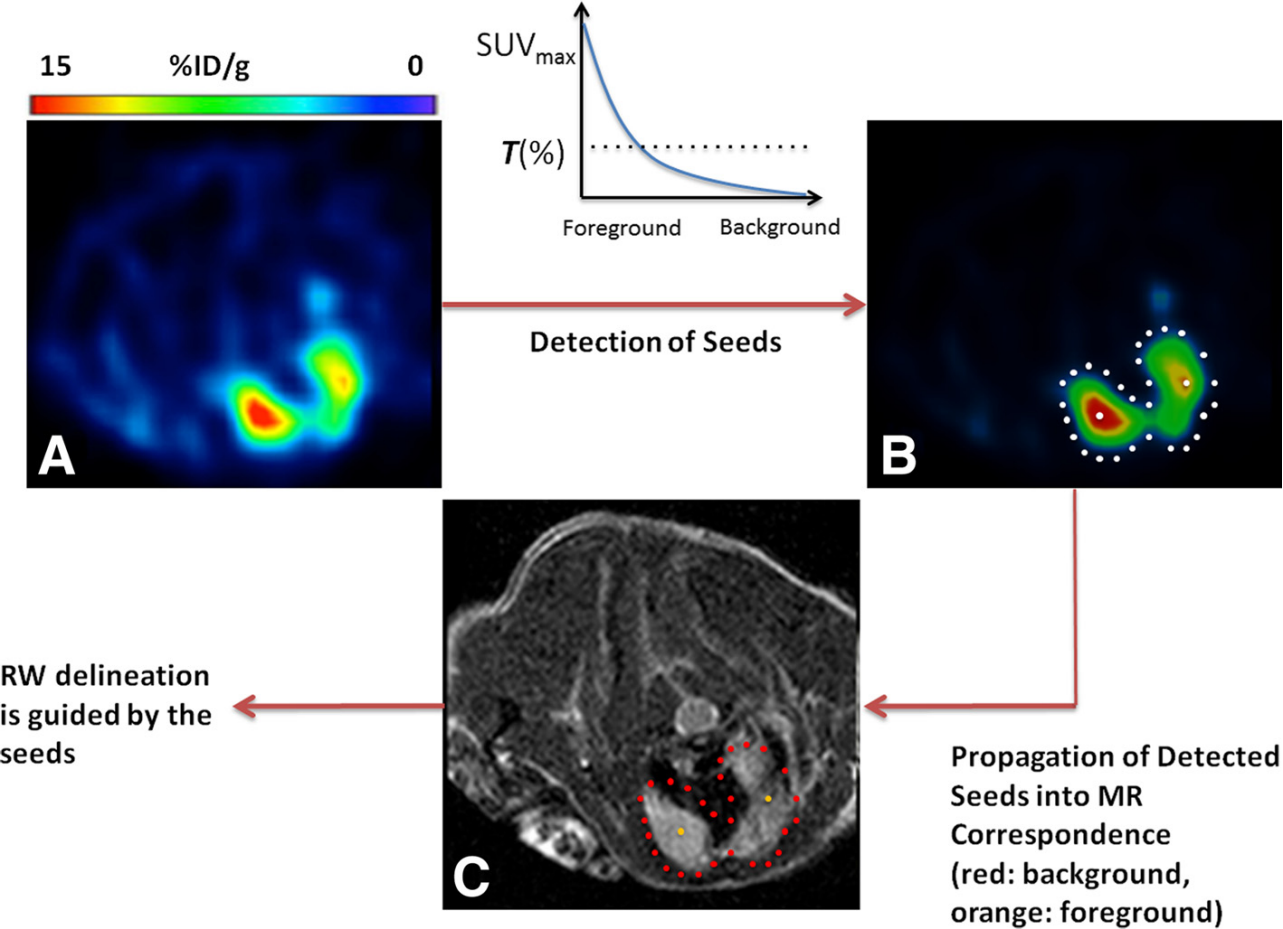
**The concepts of automatic detection of foreground and background seeds are sketched in (A to C).** Foreground seeds are located based on the encoder function (i.e., thresholding) given in **(A to B)**. Foreground seeds are allocated in the high uptake areas, and their neighbors are searched for background regions **(B)**. Foreground and background seeds are propagated into MR correspondence **(C)**. Random walk delineation is conducted by the guidance of foreground and background seeds.

For background seeds, at each voxel marked as a foreground seed, we explore its neighborhood through a 26-adjacency graph labeling algorithm in 3D (one may use 8-adjacency if the segmentation is desired in 2D) [19]. For all 26 directions starting from each foreground seed, we find locations of the very first voxels with values less than or equal to SUV_max_/*N*. Next, we add additional background seeds into the voxels lying in the spline connecting background seeds, determined in the previous step. A schematic illustration of the seeding process is given in Figure 1.

Once the seeding process is finalized, the location information of the seeds are propagated into corresponding MR images (Figure 1C). *Globally optimal* boundaries are then obtained after processing random walk segmentation with automatically determined background and foreground seeds. Further details of the proposed method is given in the next section.

#### PET-guided random walk MR image co-segmentation

We represent an image as a *graph* such that *nodes* and *edges* of the graph are defined by space elements of the image (i.e., voxels), and edges of the graph are assigned with cost values corresponding to voxel adjacency. Graph-based segmentation methods partition the nodes into two disjoint subsets representing the object and background. Suppose *G*=(*V*,*E*) is a weighted undirected graph with vertices (nodes) *v*∈*V* and edges *e*∈*E*⊆*V* ×*V*. Let an edge spanning two vertices, *v*_*i*_ and *v*_*j*_, be denoted *e*_*ij*_, and weight of an edge be defined as *w*_*ij*_. As common to graph-based approaches, edge weights are defined as a function, which maps a change in image intensity to edge weights. In particular, we use un-normalized Gaussian weighting function to define edge weights as:

(2) $$w_{\text{ij}}^{\text{MRI}}=\text{exp}(-\beta^{\text{MRI}}{\left(I_{i}^{\text{MRI}}-I_{j}^{\text{MRI}}\right)}^{2}),$$

where *I*_*i*_ indicates the intensity at voxel *i* and *β*^MRI^ represents a weighting (i.e., visibility) factor. Conventionally, the desired random walker probabilities have the same solution as the combinatorial Drichlet problem [19,20]:

(3) $$\;D[x]=\frac{1}{2}{\left(\text{Ax}\right)}^{T}C(\text{Ax})=\frac{1}{2}x^{T}\text{Lx}=\frac{1}{2}\underset{e_{\text{ij}}\in E}{\sum }w_{\text{ij}}^{\text{MRI}}{\left(x_{i}-x_{j}\right)}^{2},$$

where *x* denotes the probability (potential) assumed at each node [20]. While *C* is the diagonal matrix with the weights of each edge along the diagonal, *A* is the incidence matrix indicating combinatorial gradients, and it can be defined as:

(4) $$A_{e_{\text{ij}}v_{k}}=\left\{\begin{array}{cc} 1 & \text{if}\;i=k\\ -1 & \text{if}\;j=k\\ 0 & \text{otherwise.} \end{array}\right)$$

Furthermore, *L* in Equation 3 represents the combinatorial Laplacian matrix and can be formulated as:

(5) $$\;L_{\text{ij}}^{\text{MRI}}=\left\{\begin{array}{cc} d_{i}^{\text{MRI}} & \text{if}\;\;i=j\\ -w_{\text{ij}}^{\text{MRI}} & \text{if}\;\;v_{i}^{\text{MRI}}\;\;\text{and}\;\;v_{j}^{\text{MRI}}\;\;\text{are adjacent nodes}\\ 0 & \text{otherwise,} \end{array}\right)$$

where *v*^MRI^ is the node pertaining to the graph constructed on MRI and *d*_*i*_ is the degree of a vertex considering all edges *e*_*ij*_ incident on *v*_*i*_ and is defined as:

(6) $$d_{i}=\underset{e_{\text{ij}}\in E}{\sum }w^{\text{MRI}}(e_{\text{ij}}).$$

In random walk image segmentation, note that some of the nodes of the lattice are known (i.e., fixed, labeled), *V*_*M*_, through the seeding process, and some are not known, *V*_*U*_, such that *V*_*M*_∪*V*_*U*_=*V* and *V*_*M*_∩*V*_*U*_=*∅*. The segmentation problem in this case is basically to find the labels of unseeded (not fixed) nodes. To solve this problem, it is sufficient to solve Equation 3 through determining the critical points of the system of equations, in other words, differentiating *D*[*x*] with respect to *x* and solving the system of linear equations with |*V*_*U*_| unknowns. Solution of the system of equations yields a set of labels for unseeded nodes if every connected component of the graph contains a seed.

Since the solution of the system defined in Equation 3 is the combinatorial Dirichlet problem, random walk efficiently and quickly determines the highest probabilities for assigning labels to the pixels by measuring the ‘betweeness’ through the initial pixel of the random walk (labeled pixel) to the un-labeled pixel reached first by the random walker. A resulting probability map was used to assign foreground and background labels to the images. With this step, boundary identification of the object of interest was finalized.

### Statistical analysis

A quantitative evaluation of the segmentation algorithm was assessed using true-positive (TP) and false-positive (FP) rates showing the amount of tissue truly/falsely segmented by the proposed method. Linear regression was used to obtain the individual slope for estimated volumes, from each expert’s manual delineation. Detailed correlation analysis of the two segmentation methods (manual and automated) was also conducted by Bland-Altman analysis.

## Results

### Comparison of ^***18***^F-FDG-PET to ^***18***^F-Z _***HER2***_-Affibody-PET imaging

#### Qualitative comparison

**Figure 2:**
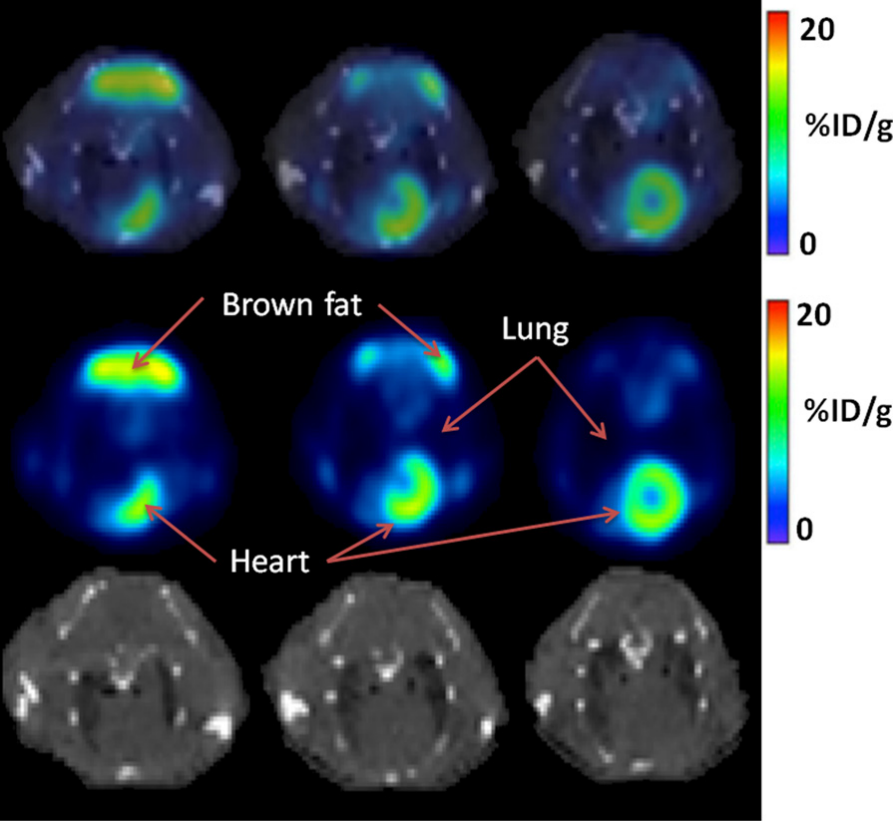
^**18**^**F-FDG-PET, CT, and fused (PET-CT) images of a representative mouse.** PET/CT (transaxial) sections of HER2-positive lung metastasis of a representative mouse, 1 h post-^18^F-FDG injection (collected 9 weeks after cell injection). First row: fused PET/CT, second row: PET, and third row: CT.

**Figure 3:**
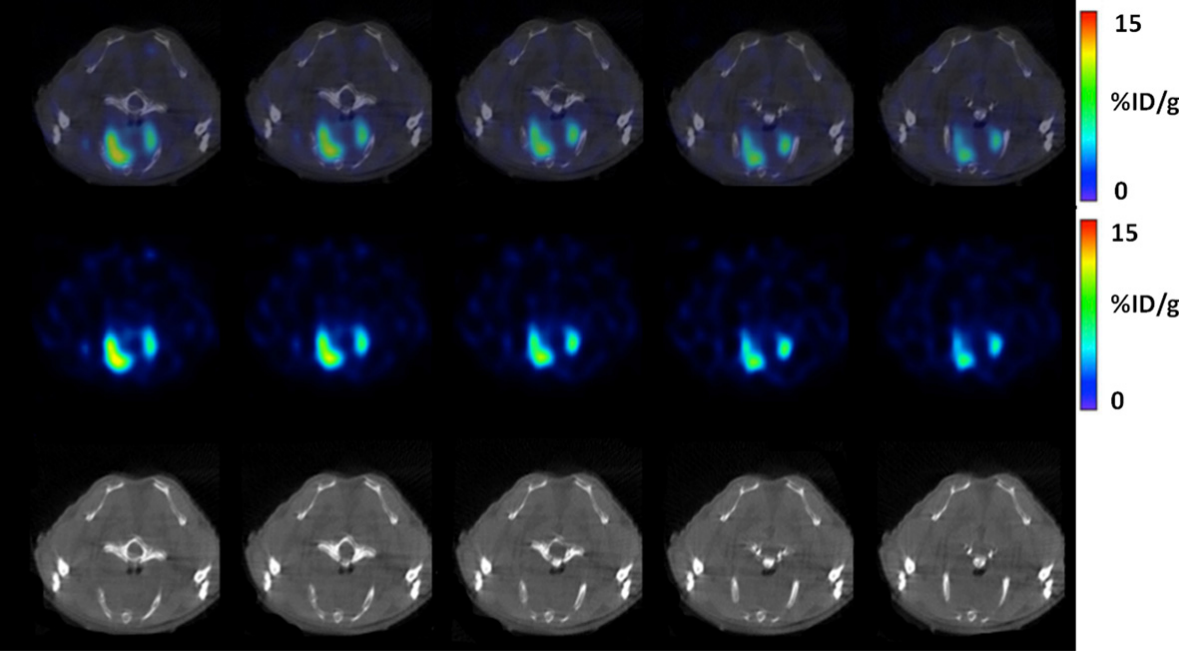
^**18**^F-Z _***HER2***_**-Affibody-PET, CT, and fused (PET-CT) images of a representative mouse.** PET/CT (transaxial) sections of HER2-positive lung metastasis of a representative mouse, 1 h after ^18^F-Z _HER2_-Affibody injection (collected 9 weeks after cell injection). First row: fused PET/CT, second row: PET, and third row: CT.

Figures 2 and 3 show representative results for qualitative analysis of PET/CT images with ^18^F-FDG and ^18^F-Z _HER2_-Affibody radiotracers, respectively. As can be seen from Figure 2, ^18^F-FDG uptake was localized in the heart, lung, and interscapular brown adipose tissues, and this lead to difficulties for localizing pulmonary metastases. Increased background activity made the evaluation of small pulmonary nodules difficult in ^18^F-FDG-PET/CT imaging, whereas pulmonary metastases were localized sufficiently well when ^18^F-Z _HER2_-Affibody was used.

#### Quantitative comparison

**Figure 4:**
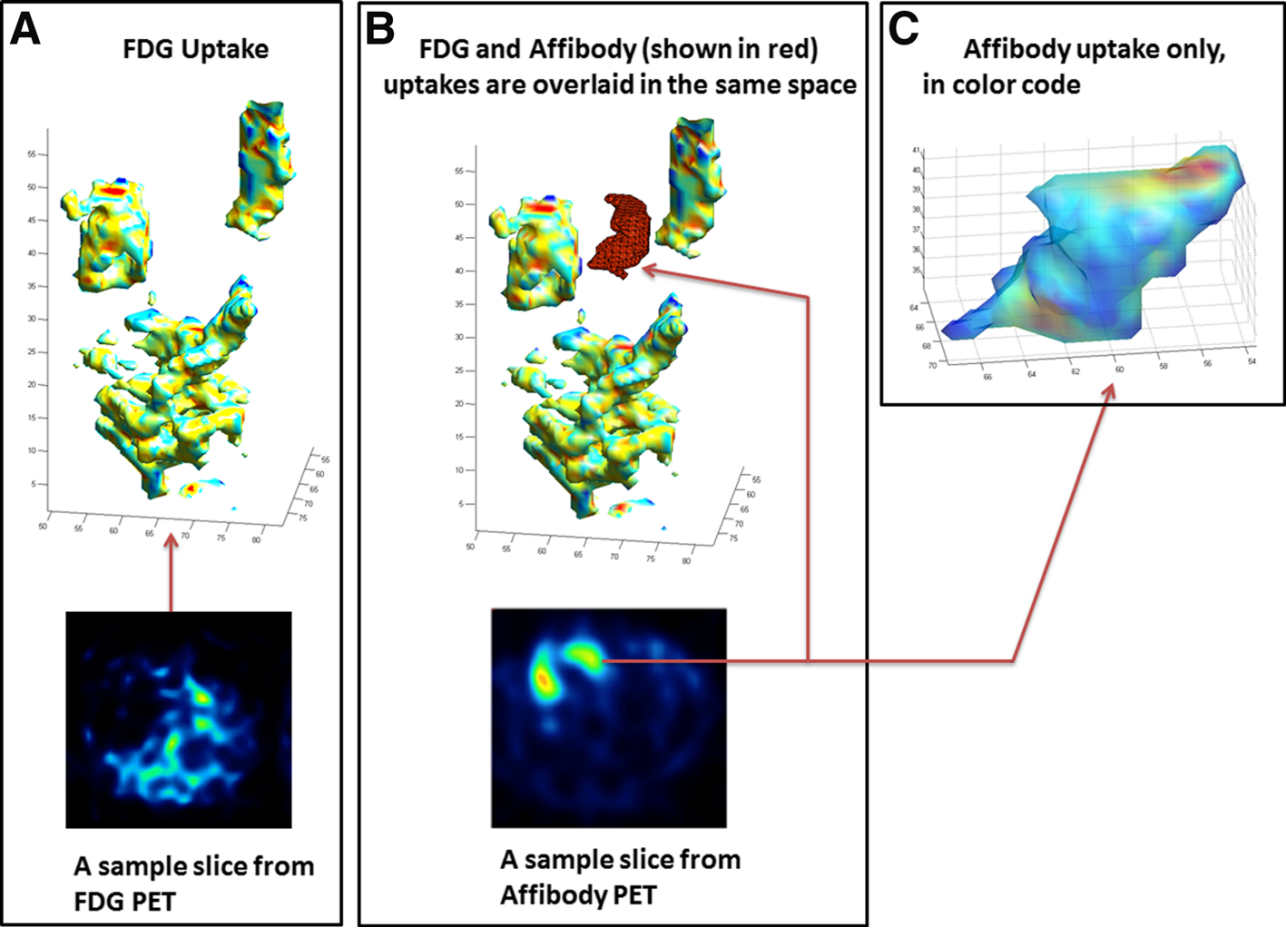
^**18**^F-FDG and ^**18**^F-Z _***HER2***_**-Affibody localization through surface renderings of segmented regions.(A)** Rendered ^18^F-FDG uptake regions of a representative mouse after segmentation. **(B)** Rendered ^18^F-Z _HER2_-Affibody uptake regions (shown in red) of the representative mouse after segmentation was overlaid with segmented FDG uptake regions. **(C)** Segmented volume due to the ^18^F-Z _HER2_-Affibody tracer localization is shown in color code.

For quantitative analysis, we automatically and roughly identified uptake regions from PET images using both ^18^F-FDG and ^18^F-Z _HER2_-Affibody, and the resulting renderings are shown in Figure 4. Note that while ^18^F-FDG radiotracer did not show specific localization of the pulmonary metastases regions (Figure 4A), ^18^F-Z _HER2_-Affibody radiotracer showed better identification of the tumor regions through segmenting the corresponding tissues in MR images (see Figure 4B,C).

#### Structural comparison

In addition, we conducted structure-based image similarity measurements to quantify the similarity of PET images using ^18^F-FDG and ^18^F-Z _HER2_-Affibody. As an image similarity metric, we used the complex wavelet-based structural similarity index (CWSSI) [21], where 1 and 0 CWSSI values indicated strong and weak structural image similarities, respectively. The similarity between those PET images was found to be 0.7838 (of CWSSI value). This indicates that without having segmentation experiments, one may show that there is a significant structural difference between PET images with different radiotracers. The differences are mainly due to the the amount of FP uptake regions in ^18^F-FDG PET images as demonstrated in the segmentation experiments. Readers are referred to [3] for a detailed comparison of ^18^F-FDG and ^18^F-Z _HER2_-Affibody on a pre-clinical basis, including detection and characterization capabilities of each radiotracer.

### Comparison of PET/MR and PET/CT through expert delineation

#### Qualitative comparison

**Figure 5:**
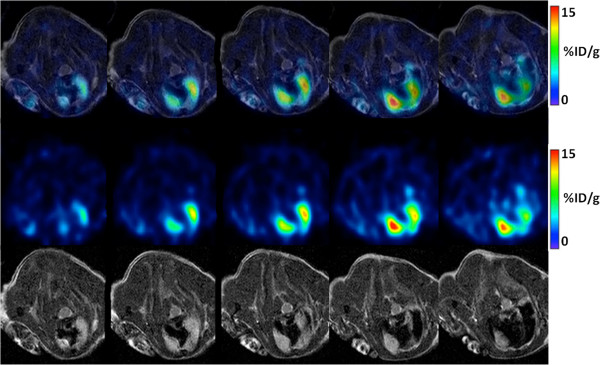
^**18**^F-Z _***HER2***_**-Affibody-PET, MRI, and fused (PET/MRI) images of a representative mouse.** PET/MRI (transaxial) sections of HER2-positive lung metastases of a representative mouse, 1 h after ^18^F-Z _HER2_-Affibody injection (collected 9 weeks after cell injection). First row: fused PET/MRI, second row: PET, and third row: MRI.

PET/MR and PET/CT examinations were feasible in all animals. As Figure 5 depicts qualitatively, MR showed higher diagnostic image quality than CT (Figures 2 and 3) due to its superior soft tissue contrast. CT had resolution limitations when differentiating tissue types (normal vs. abnormal). Fused PET/CT and PET/MRI showed correct localization of tumor regions when ^18^F-Z _HER2_-Affibody was used in PET imaging, but without PET uptake information, it was almost impossible to differentiate abnormal tissues from normal tissues in CT only (third rows of Figures 2 and 3). On the other hand, MR indicated the boundary of tumor regions to some extent without using PET information. In order to quantitatively validate this difference and to observe the limitations of the CT, we compared the spatial extent of uptake regions from PET and the anatomical correspondences of those regions from MR or CT by manual delineations provided by two expert clinicians (blinded to their drawings). Observers were presented with fused PET/CT and PET/MRI, and they randomly selected and delineated 30 lesions from all image sets. Expert clinicians also independently localized the true boundary of the lesions and then volumes and boundaries of the segmented lesions were used to evaluate observer agreement.

#### Quantitative comparison

**Figure 6:**
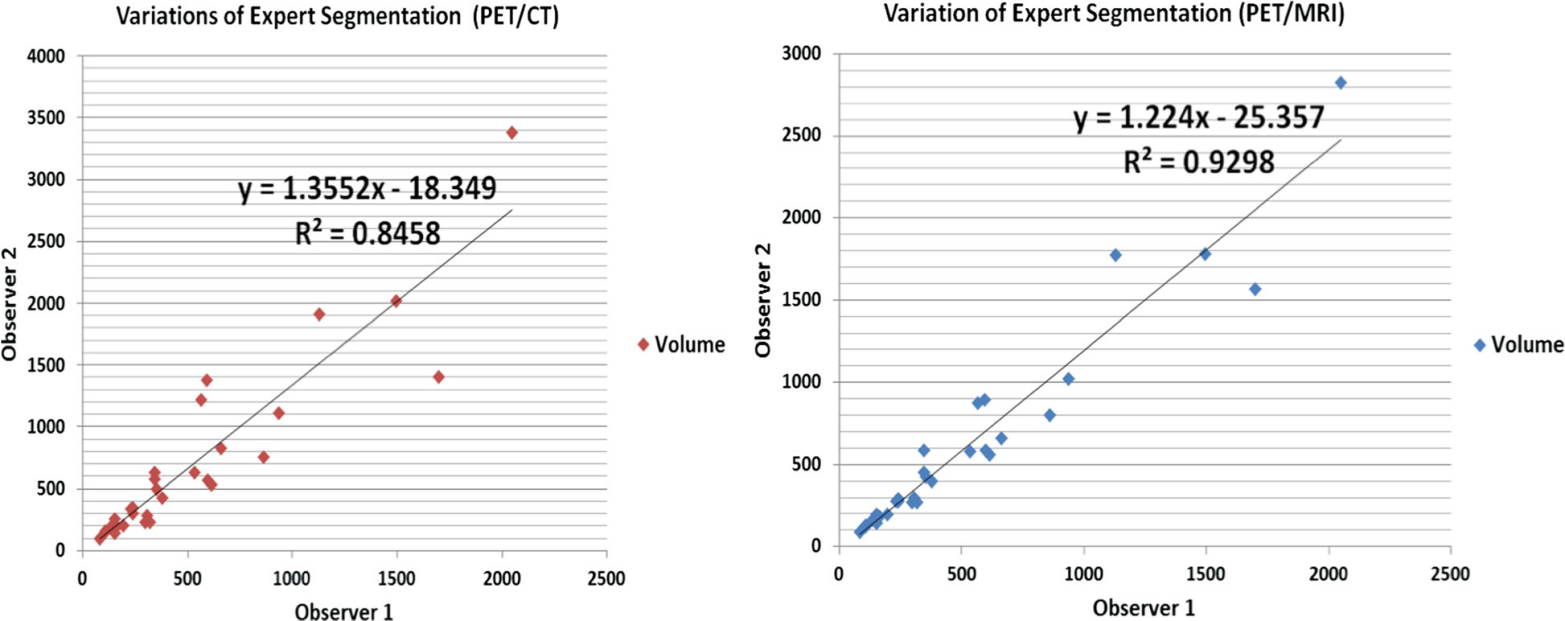
**Observer agreement rates.** Observer agreements on segmentation of 30 lesions in PET/CT (left) and PET/MRI (right) are shown. Volumes were computed as number of voxels within the segmented regions

Figure 6 shows linear regression analysis of volumes determined through manual segmentation by expert clinicians. As indicated by the correlation coefficients (*R*^2^=0.84 in PET/CT and *R*^2^=0.92 in PET/MRI), observers identified quite similar volumes in PET/MR images, but not in PET/CT. These inter-observer agreement rates suggest that MRI may be superior to CT for imaging breast cancer with metastases to the lungs in small animals. Although we emphasize the fact that MRI ‘may be’ superior to CT for imaging breast cancer with lung metastases, it is also important to note that this conclusion is valid only when lesion boundaries and accurate volumes are essential. Further validation through biopsy could potentially support this fact. Nevertheless, the proposed PET-guided anatomy segmentation in MRI is applicable to CT images as verified by the comparison experiments.

### Qualitative and quantitative evaluation of the proposed co-segmentation method

**Figure 7:**
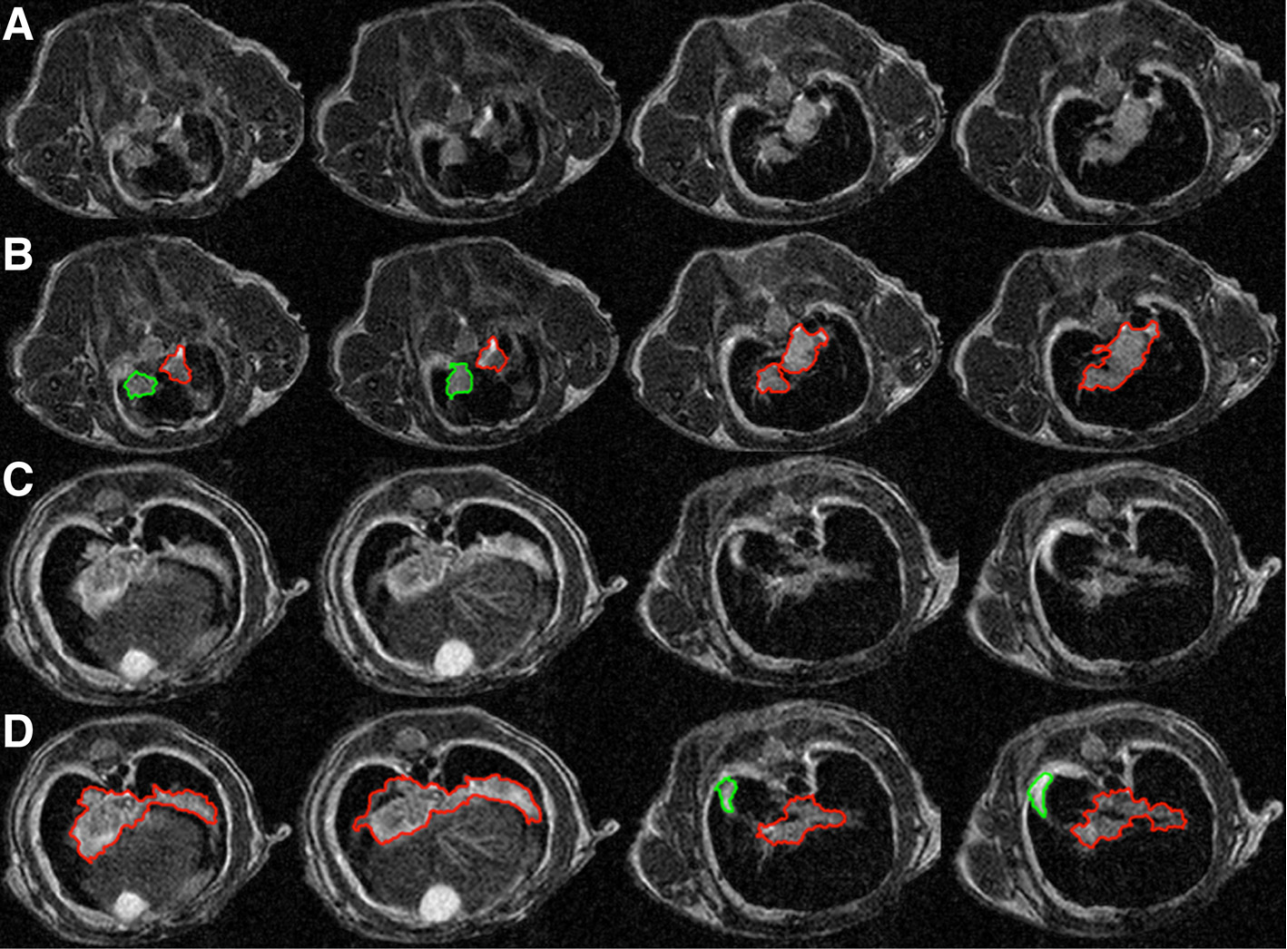
**Qualitative evaluations of the proposed segmentation technique.(A)** and **(C)** show examples of MRI axial slices from a mouse that had breast cancer. **(B)** and **(D)** show the accurately delineated pathological site using our proposed method, corresponding to slices in **(A)** and **(C)**. While connected tumor region are shown in red, disconnected regions are indicated in green

**Figure 8:**
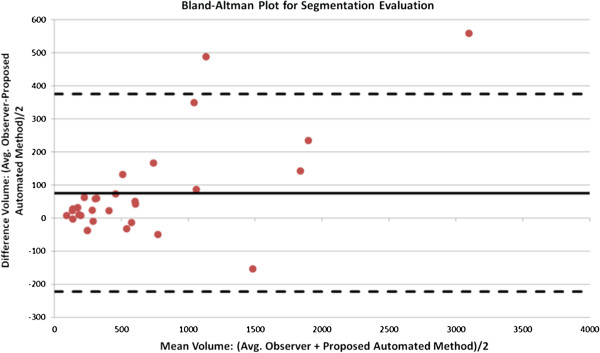
**Blant-Altman plots for automated and manual segmentation results are given.** Bias =76.5, 95% limits of the agreement = [ −222.4, 375.4]. Units are in number of voxels. Solid and dashed lines indicate mean of differences and its 95% confidence interval, respectively.

We evaluated the presented delineation technique both qualitatively and quantitatively. Figure 7 demonstrates some of the segmented slices from MR images obtained by the proposed method. For quantitative evaluation, we compared TP and FP volume fractions of the segmented tissues with the reference truth, obtained by two expert clinicians. Note that it is often the case in medical and biomedical image analysis tasks that radiologist-derived volumes are accepted as reference truths (i.e., surrogate truths) when biopsy-proven volume information is not available, as it is the case in this study. For given reference truths, it is also necessary to present inter- and/or intra-observer variations along with segmentation results for statistical validation. For a fair comparison, we averaged the performances of the two experts and reported the results in Table 1. Volumes derived by the proposed segmentation were correlated with expert-derived volumes, as mentioned in the previous subsection. After the linear regression analysis, the resulting correlation coefficient was found to be *R*^2^=0.97. Similarly, Bland-Altman plot in Figure 8 shows a strong correlation with manual segmentation results.

**Table 1:** Mean and standard deviation (SD) of TP (sensitivity) and (1 −FP) (specificity) rates within the proposed segmentation framework

**Modality**		**TP(%)**	**1-FP(%)**
PET/MRI	Mean	97.3	90.2
	SD	0.4	11.8
PET/CT	Mean	92.3	82.8
	SD	10.1	18.9
PET only	Mean	83.2	80.8
	SD	2.5	17.5
CT only	Mean	56.1	49.9
	SD	20.1	29.7
MRI only	Mean	89.5	81.4
	SD	3.1	15.8

### Comparison of the proposed segmentation method to the state of the art

**Figure 9:**
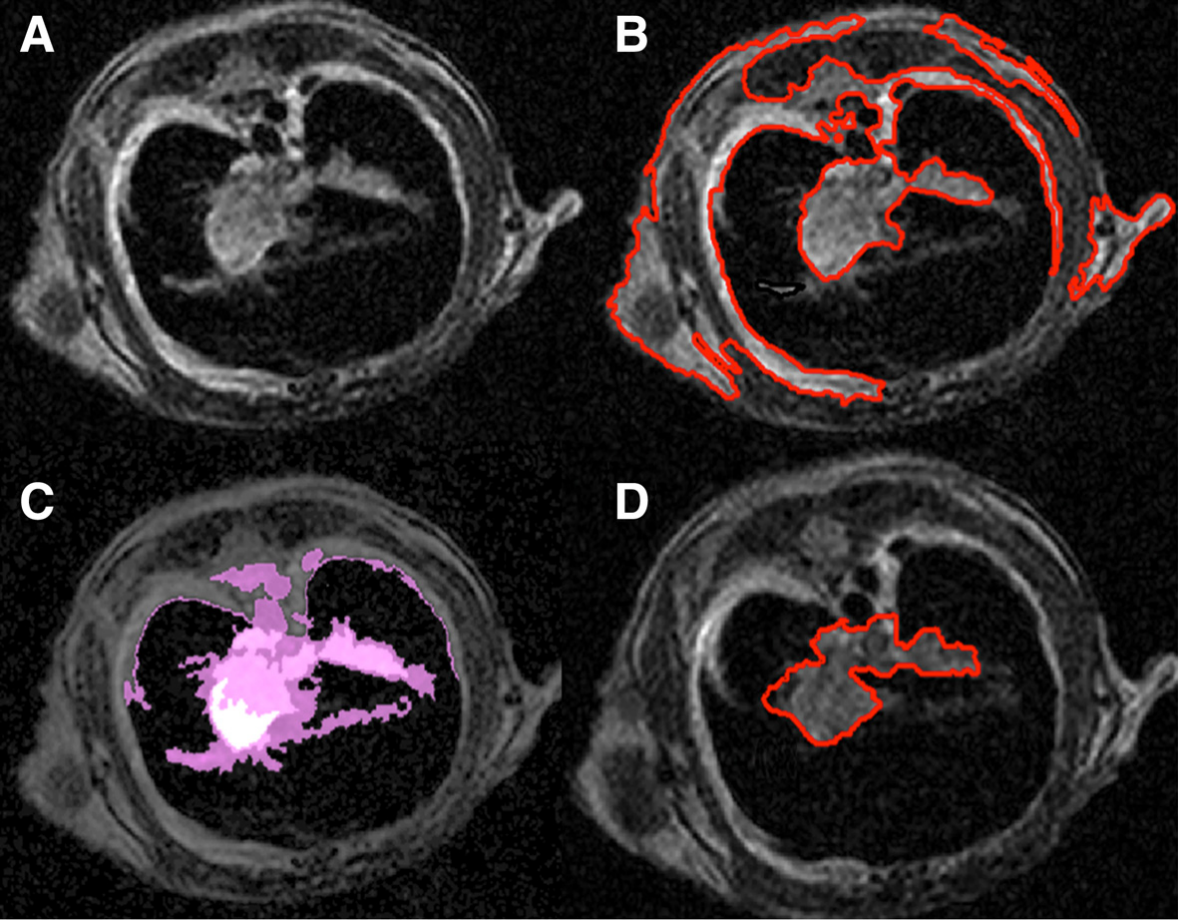
**Segmentation evaluation through comparison to the state-of-the-art methods.** For a given MRI slice **(A)**, a qualitative comparison of the proposed segmentation **(D)**, with respect to two of the state-of-the-art segmentations (i.e., region growing in **(B)** and graph cut in **(C)**), is shown. Boundary of the tumors in **(B)** and **(D)** are shown in red. Regions identified by graph cut **(C)** are shown in pink.

We compared our proposed PET-guided random walk image co-segmentation method to commonly used image segmentation methods: region growing [22] and graph cut [23]. Our proposed PET-guided MR image co-segmentation algorithm provided a precise boundary and volume identification of tumors, whereas the region growing and the popular graph cut algorithm failed to provide a precise boundary of the tumor regions. For an example MR slice demonstrated in Figure 9A, the region growing algorithm leaks into non-object territories as shown in Figure 9B, and due to the inherent noise, the algorithm lacks the ability to capture the precise boundary of the metastases region. In Figure 9C, leakage in the graph cut algorithm is inevitable owing to the complex shaped boundary of the tumor regions and highly similar intensity values of nearby objects. On the other hand, true delineation of the metastases region was obtained with the proposed method as demonstrated in Figure 9D.

Furthermore, we compared our proposed method to our previously established semi-automated method, conducted on the same animal model and published in [3]. Our proposed method provides two advantages over our previously conducted method: (1) the foreground (i.e., ROI) and background regions are identified without the help of human interaction; therefore, the proposed method herein is fully automated, and (2) the proposed method does not require an additional false-positive correction step as opposed to our previous work [3] because the proposed co-segmentation method in this study is extremely robust due to automatic identification of highly reliable background and foreground seeds. With our previously established interactive method, the total segmentation times took 20 to 25 min per animal (manual delineation can take 40 to 45 min), whereas the proposed method only takes 2 min at most.

### Robustness analysis for seed selection

**Figure 10:**
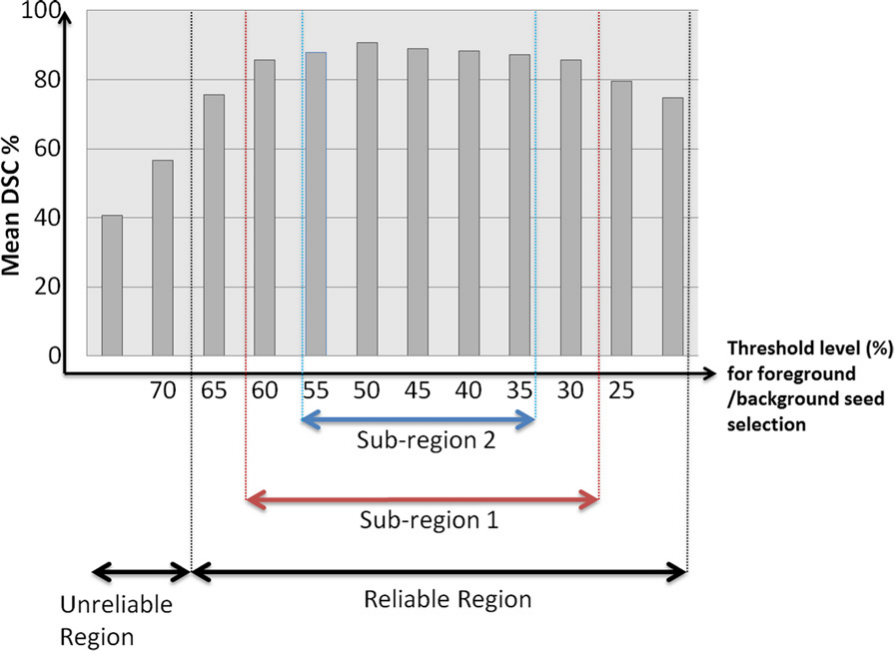
**DSC values as a function of PET thresholding parameter for background/foreground seed selection are demonstrated.** In reliable regions, DSC rates indicate accurate segmentations compared to an unreliable region, where thresholding parameter is set unrealistically high. Within reliable regions, sub-region 2 shows the highest DSC rates compared to sub-region 1; however, the DSC rates are still comparable. This fact emphasizes the robustness of the seed selection process through PET thresholding.

By considering PET guidance in our proposed framework, we are aiming to facilitate the segmentation process by constraining foreground and background regions automatically. Using an appropriate encoder function, one may easily set up localization of the foreground seeds. It is important to note that spatial positions of the foreground seeds are not affected from an encoder parameter because the foreground seeds that we identify are localized in the voxels with maximum intensity values of the uptake regions (i.e., SUV_max_). On the other hand, the background seed selection mechanism may be affected by encoder function. To analyze this effect, we conducted additional experiments with varying thresholding parameters (i.e., *N*) for the background seed selection process. Figure 10 shows the average dice similarity coefficient (DSC) rates as a function of *N* (i.e., thresholding level for PET images), where the most accurate results were obtained when *N* was selected within the reliable region (i.e., corresponding to typical thresholding values used in clinical routine). Among them, the sub-region 2 gave almost the same DSC rates and was slightly better than sub-region 1. Note that most of the thresholding values give reasonably accurate segmentation results except when *N* is set unrealistically high. Even in that case, around 60% accuracy can be observed. Note that *N*=2.5 corresponds to a 40% thresholding of PET images in practice, which is accepted as a clinical routine in several studies. With our framework, similar thresholding parameters are all lying in the reliable region of the foreground/background seed selection as demonstrated in Figure 10.

### Computational cost and parameter training

All programs used in this study were developed using C++, gcc 4.5 (Copyright (c) 2010 Free Software Foundation) on a Linux workstation, and all statistical computations were processed in R (version 2.12.2) and MATLAB (Copyright (c) 2010 Mathworks). The time required to identify background and foreground seeds from PET images and propagate the these seeds into anatomical correspondences for final guidance in the delineation took 2 min at most per animal, whereas the time required for manual identification of PET regions and corresponding boundaries in anatomical images took an average of 40 to 45 min per animal. For parameters of the graph cut algorithm, randomly selected slices from MR images with tumor metastasis were used to determine optimum parameters for background and foreground intensity priors. Similarly, mean and standard deviation of intensity values for a few tumor regions were used as a homogeneity parameter for the region growing algorithm.

## Discussion

In our work, CT to PET and MRI to PET image fusions were provided by a co-registration process in which small alignment errors may occur. Therefore, manual adjustment and qualitative judgment by expert clinicians were often required. This could possibly decrease the efficiency of a study design in terms of timing; however, the misalignment problem can be solved by hybrid imaging techniques. For instance, recently developed MRI-PET scanners are starting to be used in small-animal models [24], where no co-registration process is required since MR and PET images are obtained simultaneously with this new hybrid imaging technology.

Measuring the quality of a segmentation algorithm is the key for creating a deployable system, and it has long been a research issue how to evaluate segmentation algorithms when there is no absolute ground truth. The performance of expert observers generating segmentations of medical images has been difficult to quantify because of the difficulty of obtaining or estimating a known true segmentation for clinical data. Unfortunately, there is no ground truth available if histopathologic samples are not available. This is the main hurdle for all medical image segmentation algorithms. Instead, scientists use manually segmented structures and compare those structures with algorithm-generated segmentations in terms of overlap or boundary differences. Currently, manually creating reference truths is the state-of-the-art segmentation evaluation and development strategy for the medical image processing and analysis field. Although we followed the state-of-the-art evaluation metrics for image segmentation in our work, we would like to note a very recent work of Kohlberger et al. [25], where authors presented a generic learning approach based on a novel space of segmentation features, which can be trained to predict the overlap error and dice coefficient of arbitrary organ segmentation without knowing the ground truth delineation. Although measuring the quality of a segmentation produced by an algorithm is a long-standing research topic, arguably, it may be possible in the near future to evaluate image segmentation algorithms without having a reference truth.

^18^F-FDG-PET/CT imaging has emerged as a clinical cornerstone in many diseases, including oncology, infection, and inflammation. Because ^18^F-FDG is a non-specific marker of cell metabolism, which may be elevated in tumor growth as well as in immunologic reactions, the search for more specific markers of disease continues. In our study, we demonstrated quantitatively that ^18^F-FDG was not as strong of a predictor as the ^18^F-Z _HER2_ Affibody radiotracer when quantifying HER2-positive breast cancer lesions. We did not re-iterate the invention of tumor-specific antigen; however, we did provide a suitable computational platform that clinicians and researchers need for measuring anatomical volumes and spatial extent of the functional information of tumors accurately, robustly, and longitudinally - and within seconds (for heightened efficiency). The importance of assessing lesion volumes, as well as measuring cellular behavior within the lesions, accurately and efficiently arises from the need to perform *in vivo* monitoring of disease progression versus regression in response to treatment in clinical and preclinical (animal-based) studies. Characterization of lesions - in terms of volumes and molecular cellular characteristics - potentially assists staging, which predicts how aggressive the lesion will be in causing future disease in the patient.

MRI may be a useful alternative to CT in hybrid PET imaging. Although MRI has the benefit of contributing no ionizing radiation to the patient’s exposure (unlike CT), the use of MRI with PET (instead of PET/CT) is still an ongoing subject of intense investigation to properly diagnose and measure lesions. There are currently numerous studies in the literature comparing MRI with CT for visualization and quantification of the lungs, and CT is seemingly far superior at visualizing the lungs and organs in the chest cavity. Although this is true for many disease models within the lung anatomy, it does not change the effectiveness of our method when segmenting breast cancer metastases in our small-animal model using MRI. Our proposed method can also be used for PET-CT hybrid imaging, as demonstrated in our previous co-segmentation studies [17,18].

Although it would be useful to investigate the functional characterization of the breast tumors (i.e., benign and malignant) when the reference standard (i.e., biopsy specimens) is available, this subject is outside the scope of this paper. Nevertheless, potentially useful quantitative functional information can also be obtained with our proposed quantification framework such that precise functional characterization of the metabolic activities (i.e., based on the SUV of uptake distribution) may be possible with the accurate co-segmentation technique we proposed herein.

## Conclusions

In the present study, we quantitatively and qualitatively compared both molecular imaging agents used in PET imaging (^18^F-FDG vs. ^18^F-Z _HER2_-Affibody) and structural imaging modalities (CT vs. MRI). We determined that the ^18^F-Z _HER2_-Affibody radiotracer used in PET imaging, when combined with corresponding MR images, provided the most suitable platform for robust and accurate volume quantification of HER2-positive breast cancer lesions in small-animal models. Because the proposed method can effectively merge and optimize information from both anatomical and functional images, our method holds a real potential for identifying new image-based markers. This computational aid may improve the efficiency and cost-effectiveness of radiology and nuclear medicine workflows.

## Abbreviations

CT: computed tomography; CI: confidence interval; CWSSI: complex wavelet-based structural similarity index; DSC: dice similarity coefficient; FA: flip angle; FDG: fluorodeoxyglucose; FFE: fast field echo; FOV: field of view; FP: false positive; MRI: magnetic resonance imaging; PET: positron emission tomography; ROI: region of interest; SD: standard deviation; TE: echo time; TP: true positive.

## Competing interests

The authors declare that they have no competing interests.

## Authors’ contributions

GK-M designed the lab experiments including the imaging of mice. GK-M, UB, and DJM conducted the research for quantification using imaging markers. UB developed the segmentation methods for quantitative and longitudinal analysis of small-animal images. UB wrote the software for segmentation evaluation test for inter- and intra-operator variation derivation. UB conducted all statistical tests, and two bio-statisticians independently checked those tests. The manuscript was written by GK-M, UB, and DJM. All authors read and approved the final manuscript.
